# Evolution of oxidative stress markers in livers of ducks during force-feeding

**DOI:** 10.1038/s41598-022-27271-y

**Published:** 2023-01-19

**Authors:** Herve Remignon, Pierre Burgues

**Affiliations:** 1 Toxalim (Research Centre in Food Toxicology), Université de Toulouse, INRAE, ENVT, UPS, 31300 Toulouse, France; 2 INP-ENSAT, Université de Toulouse, 31320 Castanet-Tolosan, France

**Keywords:** Gastrointestinal models, Mechanisms of disease

## Abstract

Mule ducks have been force-fed to develop a hepatic steatosis, also called “foie gras”, which is similar to the non-alcoholic fatty liver disease (NAFLD) described in humans and mammals. However, in hepatic steatosis resulting from force-feeding of ducks, very little is known about the fine biochemical events that occur due to the enormous and very rapid increase in total lipids that mainly accumulate in hepatocytes. To begin to reduce this lack of knowledge associated with the development of this specific hepatic steatosis, liver samples were taken at different times to follow the overall biochemical transformation of the liver as well as different markers of oxidative stress, hypoxia and apoptosis. The results indicate that the lipid content increases rapidly in the liver throughout the force-feeding period while the protein content decreases. The amount of hydroxyproline remains constant indicating that no liver fibrosis develops during the force-feeding period. On the contrary, all the tested biomarkers of cellular oxidative stress increase rapidly but without any visible disorder in the coordination of paired activities. At the same time, hypoxia-inducible factors also increase indicating that a hypoxia situation is gradually occurring in hepatocytes. This leads, in addition to the lipotoxicity induced by the accumulation of lipids, to an increased number of liver cells to enter into apoptosis. A relative variability in the level of these cellular responses was also observed indicating that, probably, certain animals support the development of this steatosis differently. This leads us to imagine that the physiological status of these birds may differ widely for reasons that remain to be clarified.

## Introduction

The production of “foie gras” is traditional in France and is typical of French gastronomy which has been included on UNESCO’s list of Intangible Heritage of Humanity since 2010. To obtain this food product, waterfowl (either geese or ducks) are force-fed until they develop a liver steatosis. The metabolic mechanisms associated with the development of this hepatic steatosis are very similar to those described in human as non-alcoholic fatty liver disease (NAFLD) and/or non-alcoholic steatohepatitis (NASH). In short, it is the daily consumption of large quantities of carbohydrates that places the liver of force-fed ducks in a permanent state of synthesis of lipids which are only partially exported into the peripheral tissues, mainly the abdominal and subcutaneous adipose tissues. Numerous studies^1–6^ have been conducted to study the development of this hepatic steatosis in force-fed ducks and mainly indicate that the de novo lipogenesis is dramatically increased while other hepatic metabolisms are also modified (proteolysis and proteogenesis, lipids exportation, carbohydrates metabolism, and programmed cell deaths, etc.). However, none have focused on the oxidative stress that could be at the origin of those large metabolic dysfunctions of the hepatic cells.

Even in humans, NAFLD remains a complex and multifactorial disease associated with a myriad of genetic, epigenetic, and environmental factors, and its pathogenesis is still not entirely clear^7^. Hepatic steatosis increases liver lipogenesis but hinders the breakdown of free fatty acids (FFA). Therefore, lipid accumulation sensitizes the liver to induce inflammation and cell death by pathogenic aggression that promotes oxidative stress, culminating in NASH and fibrosis. Oxidative stress is thus considered to be the main contributor to liver damage and disease progression in NAFLD^8^. According to^9^, this first hypothesis has been subsequently revised in a “multiple parallel-hit” model in which, in the presence of a significant accumulation of fat in hepatocytes and systemic and hepatic insulin resistance, multiple simultaneous alterations lead to an imbalance between the antilipotoxic protection system of the liver (mitochondrial ß-oxidation) and the free radical production in gut and adipose tissue, resulting in endoplasmic stress, oxidative stress, and hepatocyte apoptosis.

For the present study, we induced the development of hepatic steatosis in ducks by force-feeding them for 10 days. Then, we measured throughout the development of steatosis several hepatic biomarkers to follow its biochemical transformation possibly associated with a restricted supply of oxygen, the development of cellular oxidative stress and finally its possible transition to an apoptotic state. The objective of the study was therefore to draw up an initial overview of the oxidative stress that can occur in the hepatocytes of force-fed ducks and to possibly associate it with the high accumulation of lipids associated with a restricted supply of oxygen and resulting in an increase in apoptotic events.

## Material and methods

### Animals and livers sampling

A flock of about 1000 male mule ducks (Caïrina moschata x Anas platyrhynchos) was reared for 12 weeks, from hatching, according to standard commercial rules. At 12 weeks of age, 12 birds (live weights similar to the whole flock mean live weight) were randomly selected and slaughtered to constitute the group of ducks at baseline (B) before the beginning of force-feeding. The remaining birds entered in the force-feeding program for 20 meals (twice a day during 10 days as farmers usually do) according to a standard force-feeding program based on moistened corn flour (97.5% of corn). At the beginning of the force-feeding period, ducks received a quantity of 225 g of dry flour/meal, which was progressively increased to a final value of 510 g for the last meal. As a whole, during the force-feeding period, ducks ingested an average quantity of 7.0 kg of feed. After 5 meals of force-feeding, 12 animals were randomly (similarly than described upper for B ducks) selected and slaughtered to constitute the group of BFF (Beginning of Force-Feeding) ducks. The remaining birds continued the force-feeding program for 15 supplementary meals. At that time, all the birds were slaughtered and 24 of them were selected to constitute the group of EFF (End of Force-Feeding) ducks. At this final step, because that data was available for the whole flock, the selection of the EFF birds was done to represent the observed variability in fatty livers weights (mean ± SD = 571 ± 114 g, CV% = 20). At each step, (B, BFF, EFF) ducks were slaughtered approximately 11 h hours after the last meal in a commercial slaughter-house according to its standardized operations of slaughtering (electronarcosis, bleeding, scalding, plucking). At the end of the slaughtering line, 20 min post mortem, livers were harvested and weighed. Then, 50 g of tissue were collected from the median lobe and directly frozen in liquid nitrogen before storage at − 80 °C.

All the biochemical measures were performed in duplicate after the grinding of the tissue in liquid nitrogen.

### Gross biochemical composition of livers

The dry matter (DM) content was determined by desiccation of crushed livers in an oven at 105 °C for 24 h^10^. The total lipids content was measured according to^11^ after extraction with chloroform:methanol (2:1, v/v). The total proteins content was determined according to the procedure described by the manufacturer (Pierce™ BCA protein assay kit) after an extraction with a phosphate buffered saline solution. The hydroxy-proline (OH-Pro) content was determined according to^12^ on the delipidated and dry residue obtained after the total lipids extraction.

### Oxidative status

GSH/GSSG analysis: The reduced to oxidized glutathione ratio (GSH/GSSG) was determined according to the protocol described by the manufacturer (catalog #: 239709, Abcam, Cambridge, UK).

The activities of the superoxide dismutase (SOD, catalog #: 19160, Sigma, St Louis, MO, USA) and catalase (Cat, catalog #: KB03012, BioQuoChem,Llanera-Asturias, Spain) enzymes were determined according to the procedures described by the manufacturer.

Results are expressed in U/mg of proteins.

### ELISA tests

HIF1α (Hypoxia Inducible Factor 1 Alpha), HIF2α (Hypoxia Inducible Factor 2 Alpha), GPX1 (Glutathione Peroxidase 1) and NQO1 (Nicotinamide Adenine Dinucleotide (Phosphate) Hydrogen Dehydrogenase (Quinone) 1) contents were determined with ELISA tests by using assay kits from MyBioSource (San Diego, CA, USA) according to the manufacturer’s protocols.

Results are expressed in pg/mg of proteins (GPX1, HIF1α, HIF2α) or in ng/mg of proteins (NQO1).

### Caspases activities assays

Activities of caspase-3, caspase -8 and caspase-9 enzymes were colorimetrically estimated via the CaspACE testing framework acquired from Biovision (Mountain View, CA, USA) in respect to the producer’s guidelines. For each enzyme, the total activity of a pool of liver extracts composed with all the available samples was measured concomitantly with the individual samples. Then, the measured activity of this pool was arbitrary set to a value of 100 and the activity of each individual sample was expressed as a percentage of this value to finally indicate the enzyme activities in arbitrary units.

### Statistics

Statistical analyses were performed with the SAS software, version 9.4 of the SAS System for Windows. Analysis of variance (ANOVA) were performed with the General Linear Model (Proc GLM) completed with the Student–Newman–Keuls’ post-hoc test to compare the means of each groups. Values are expressed as mean ± standard deviation (SD). We set the significant level at p < 0.05.

### Ethics approval

Ethical review and approval were not required for this animal study because we collected samples from a commercial slaughter house after the regular operations of anesthesia, bleeding, scalding, plucking and evisceration. We do not manipulate live animals and no specific experimental manipulations were performed during the time of rearing. All the samples we harvested were commercial ones, ready to be purchased by consumers.

## Results and discussion

As a whole, the rearing and the force-feeding programs took place without any noticeable events (mortality < 1.5%). As reported in the Table 1, after the 5 first meals of the force-feeding program (period B to BFF), the liver weights increased by 1.8 (average meal gain = 25 g) while it increased by 2.3 during the rest of the force-feeding time (period BFF to EFF, average meal gain = 24 g). This huge increase in liver weight was expected because the feeding program delivered very large quantities of corn which contain mainly carbohydrates (62% of starch). Those sugars are easily absorbed and brought to the liver where they are mainly metabolized in fatty acids by the de novo lipogenesis pathway and stored as triglycerides. This increase of the liver weight during force-feeding has already been largely documented in previous studies^2,3,13^.

**Table 1:** Chemical composition of the analyzed livers harvested before (B), at the beginning (BFF) or at the end (EFF) of the force-feeding period.

	B	BFF	EFF	RMSE	p
Liver weight (g)	150^c^ ± 32	276^b^ ± 43	632^a^ ± 120	89.70	***
Dry matter (% raw tissue)	34.08^c^ ± 1.23	45.29^b^ ± 1.46	69.34^a^ ± 3.59	4.77	***
Lipids (% of raw tissue)	4.92^c^ ± 1.44	23.49^b^ ± 1.85	52.91^a^ ± 6.48	2.74	***
Proteins (% raw tissue)	12.7^a^ ± 0.94	6.23^b^ ± 1.21	3.09^c^ ± 0.58	0.87	***
OH-Pro (mg / g delipidated and dried tissue)	0.16^a^ ± 0.05	0.11^b^ ± 0.03	0.15^a^ ± 0.06	0.05	*

*RMSE* root-mean-square error, *OH-Pro* hydroxyproline.

Values are means ± SD. Means with the same superscripts ^(a, or b, or c)^ are not different (p < 0.05).

*** = p < 0.001, * = p < 0.05.

During the force-feeding period, the dry matter and lipid contents dramatically increased while the protein one decreased. Those variations in the gross biochemical composition of the livers are conform to what were expected and simply traduce the huge accumulation of lipids, and the relative dilution of proteins, during the development of hepatic steatosis^1,4,6,14^ induced by the force-feeding. Considering the duration of the gavage period and the age of the animals between stages B and EFF, we assume that all the changes observed in the chemical composition of the liver are mainly due to the gavage and not to the increase in l age of the ducks (+ 10d for ducks 12w old). This is consistent with what has already been reported by^2,3,6,14^.

Even if the OH-Pro content of the livers was different from BFF to B and EFF groups (p < 0.05, Table 1), no big variation in the hepatic OH-Pro content was observed. This indicates that, despite its high intensity, the duration of the force-feeding is not sufficient to induce a significant increase in the development of the hepatic connective tissue. On the contrary, in mouse, during the shift from non-alcoholic fatty liver disease (NAFLD) to non-alcoholic steatohepatitis (NASH), an increase in OH-Pro content of the liver was reported^15,16^ and considered as a sign of the development of a fibrosis characteristic of the NASH state. To our knowledge, it is the first time that hydroxyproline content of the liver was determined during the development of the hepatic steatosis in force-fed ducks. Our results indicate that no NASH occurs during that period even if a large quantity of lipids accumulates in hepatocytes. This is probably due to the short time (10 d) of development of the hepatic steatosis in force-fed ducks which is not enough to generate a fibrosis per se.

**Figure 1:**
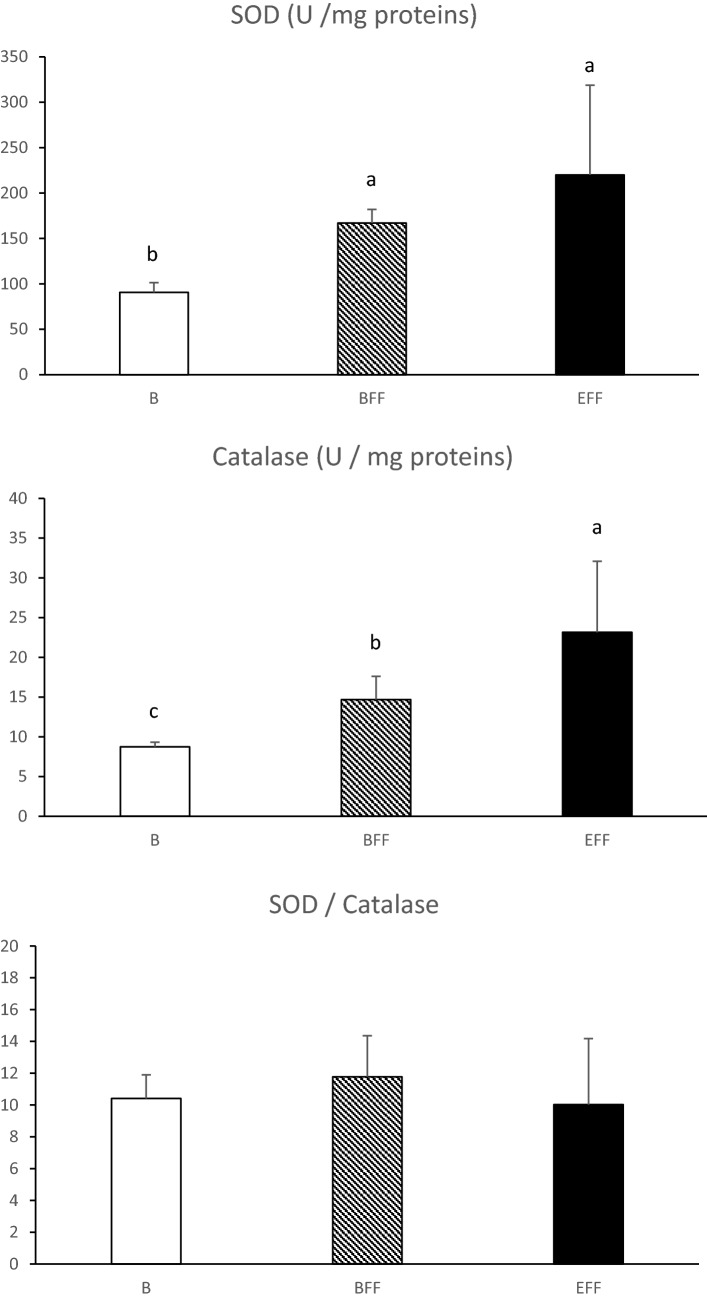
Activities of superoxide dismutase (SOD) and catalase enzymes in livers collected before (B) or at the beginning (BFF) or at the end (EFF) of the force-feeding period. Values are means ± SD. Bars with different letters differ (p < 0.05).

**Figure 2:**
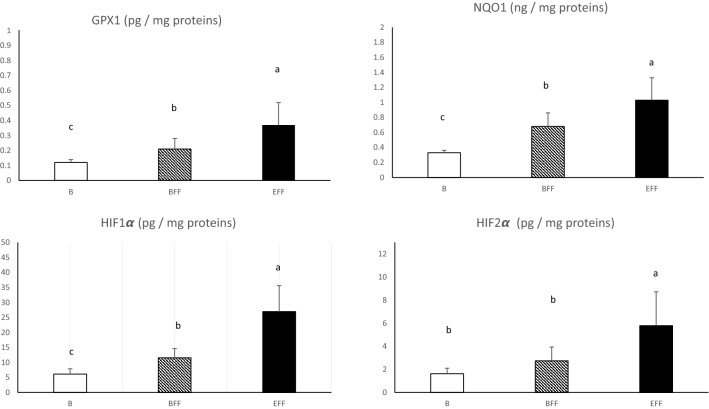
Biochemical contents of glutathione peroxidase 1 (GPX1), nicotinamide adenine dinucleotide (phosphate) hydrogen dehydrogenase (quinone) 1 (NQO1), hypoxia inducible factor 1 alpha (HIF1α) and hypoxia inducible factor2 alpha (HIF2α) in livers collected before (B) or at the beginning (BFF) or at the end (EFF) of the force-feeding period. Values are means ± SD. Bars with different letters differ (p < 0.05).

The development of the hepatic steatosis is due to the imbalance between the capacities of synthesis and export of lipids by hepatocytes. This leads to a high accumulation of newly synthesized lipids despite the increase in the export of these same lipids by lipoproteins^17,18^. This is facilitated by the fact that corn contains few lipotropic factors such as choline or methionine and a lot of vitamins B8 (biotin) that promotes the synthesis of fatty acids. According to^19^, one of the major issues with this imbalance in lipids metabolism is a subsequent metabolic stress due to the accumulation of free fatty acids (FFAs), which serve as a substrate to generate lipotoxic species that cause endoplasmic reticulum stress and produce reactive oxygen species (ROS). Those ROS are highly toxic for the cell and it tries to eliminate them with several mechanisms. Among them the coupling activity of superoxide dismutase (SOD) and catalase (Cat) is efficient to neutralise the superoxide ion O_2_^-^, one of the most powerful ROS. In the present study, SOD and Cat activities rapidly increase at the beginning (BFF) and all along the force-feeding period (Fig. 1). This indicates that force-feeding induced rapidly an oxidative stress in liver cells but the constancy of the SOD/Cat ratio also indicates that no major dysregulation of these coupled activities is present. In different pathological^20,21^ or feeding^22^ or environmental conditions^23^, the activities of SOD and Cat enzymes also increase to reduce the oxidative stress and are therefore considered to be one of the predominant antioxidant mechanisms in duck hepatocytes. Other enzymatic antioxidant systems (GPX1 and NQO1) activities are presented in Fig. 2. NQO1 removes quinone from biological systems through a detoxification reaction. This reaction oxidises the substrate without the formation of damaging semiquinone and oxygen free radical species^24^. GPX1 acts in combination with reduced glutathione (GSH) to catalyze the reduction of H_2_O_2_ in H_2_O and oxidized glutathione (GSSG). In this study, both GPX1 and NQO1 increase significantly along the force-feeding period. This supports the idea that from the beginning until the end of the development of the steatosis, hepatocytes have to face an important oxidative stress and react by enhancing their antioxidant activities.

**Figure 3:**
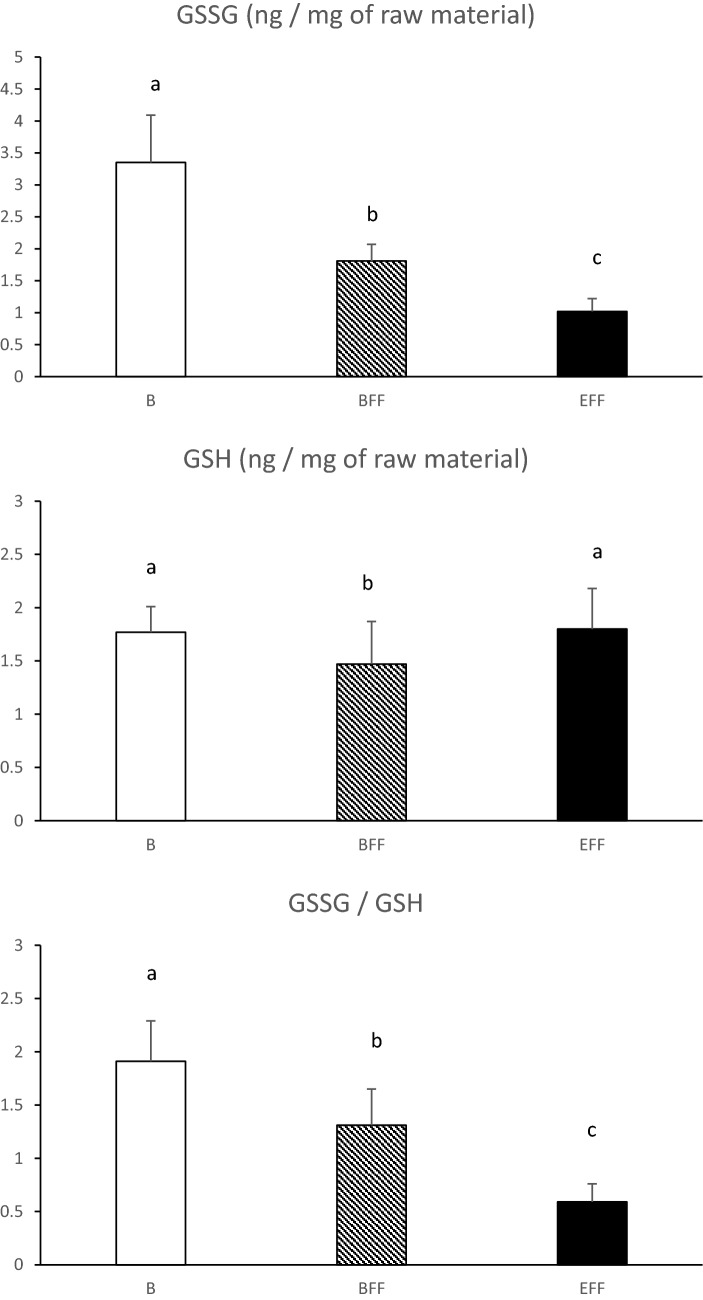
Values of oxidized glutathione (GSSG) and reduced glutathione (GSH) contents in livers collected before (B) or at the beginning (BFF) or at the end (EFF) of the force-feeding period. Values are means ± SD. Bars with different letters differ (p < 0.05).

Glutathione is a small tripeptide which is present in all cells. Reduced glutathione (GSH) plays critical roles in protecting cells from oxidative damage and maintaining redox homeostasis^25^. GSH is considered the most important intracellular antioxidant^26^. It allows the reduction of ROS thanks to the action of glutathione peroxidases (GPx) which allows the transition from the reduced form (GSH) to the oxidized form (GSSG). The GSSG/GSH ratio is therefore a good indicator of good cellular health and a decrease in its value is a sign of major cellular dysfunction^27,28^. The cell keeps a permanent control on the GSSG/GSH ratio and in a state of oxidative stress, the consumption of GSH by GPx increasing, the cell activates several pathways to renew the quantity of GSH. The increased glutathione redox status is a compensatory mechanism in response to the increased oxidative burden due to the development of steatosis. In this study (Fig. 3), the level of the GSH content remained nearly constant while GSSG and GPX1 contents varied in opposite directions from the beginning to the end of the force-feeding period. During the development of the steatosis, the accumulation of lipids in hepatocytes induces an increase in the activity of the ß-oxidation pathway and consequently a higher leak of electrons which link to the oxygen and generate ROS. If the level of response for producing more antioxidant molecules is not sufficient, excessive ROS induce strong dysregulations of mitochondrial functions^29^. According to^30^, in humans with severe NAFLD or NASH syndromes, the levels of hepatic SOD, catalase and GSH are low because the level of ROS is too high and exceeded the capacity of regulation of the hepatocytes. In the present study, these markers increase throughout the force-feeding period but ultimately do not fall which may indicate that hepatocytes are still able to cope with the rapid development of steatosis despite the observed increase in liver oxidative stress. The effective protective role of cellular antioxidants has also been suggested by^4^ who measured no increase in TBARS (thiobarbituric acid reactive substances) values in livers of different weights from force-fed ducks.

HIF1α and HIF2α are protein transcription factors involved in the response to initial or acute and delayed or chronic hypoxia, respectively^31^. In this study, HIF1α increased rapidly from start and throughout the force-feeding period while HIF2α only increased from BFF to EFF (Fig. 2). These increases indicate that during the period of force-feeding, the livers encountered hypoxic conditions that could be related to physical disability due to liver enlargement and circulatory problems linked to the elevated lipemia typically associated with the development of the hepatic steatosis. According to^32^, an increase in ROS can result from a reduction of an oxygen supply efficiency. According to^33^, in mammals, impaired hepatic metabolism and tissue remodeling seen in fatty liver disease disrupt hepatic oxygen homeostasis, resulting in severe hepatic hypoxia. Reference^34^ also reported that HIF-2 is overexpressed in humans or mice developing liver damage steatosis of various origins, which leads to a hypoxic microenvironment and mitochondrial dysfunctions. Then, it must be concluded that throughout the force-feeding period, livers are submitted to increased hypoxic situations that will favor the development of the observed cellular oxidative stress.

**Figure 4:**
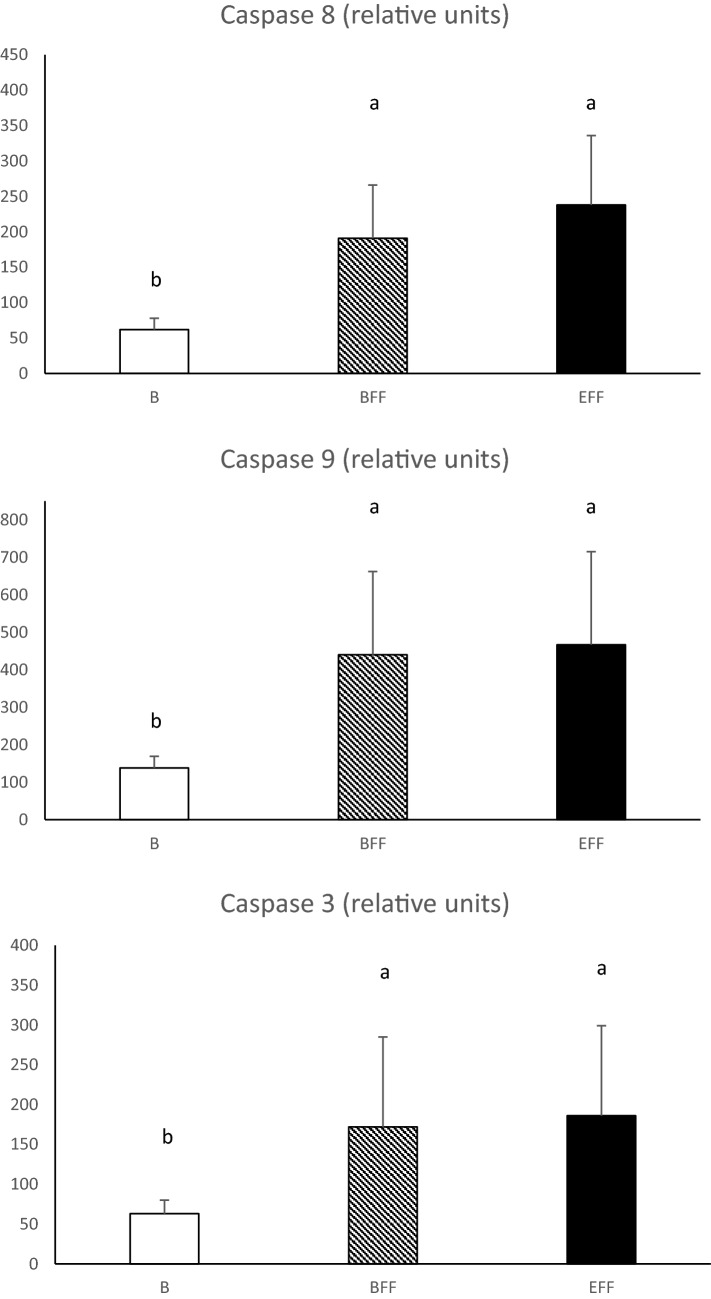
Caspase-3, caspase-8 and caspase-9 relative activities in livers collected before (B) or at the beginning (BFF) or at the end (EFF) of the force-feeding period. Values are means ± SD. Bars with different letters differ (p < 0.05).

Caspase-3 is considered as a key effector enzyme in inducing cell apoptosis. Caspase-8 and caspase-9 are initiator caspases of extrinsic and intrinsic apoptosis, respectively^35^. As reported in Fig. 4, caspase-8, -9 and -3 have a low level of activity in livers from B ducks. On the contrary, after 5 meals (BFF stage) or 20 meals (EFF stage) of force-feeding, activities of all the studied caspases significantly increase. This indicates that the development of the observed hepatic steatosis induced by the feeding program is associated with the development of apoptosis in the liver. This increase was previously reported in the liver of force-fed ducks^4,5,36^. According to^37,38^, the production of reactive oxygen species, and the development of oxidative stress and endoplasmic reticulum stress are involved in the progression of apoptosis associated with the activation of NAFLD/NASH. Thus, in the present study, the hypoxic and oxidative environments of hepatocytes in force-fed ducks might also be involved in the observed onset of apoptosis development, while evidence for the transition from NAFLD to NASH is not still visible. Moreover, given that both activities of caspase-8 and -9 increased during the force-feeding period, none of the extrinsic or intrinsic activation pathways can be favored to explain the development of apoptosis.

## Conclusion

During the development of force-feeding induced hepatic steatosis in ducks, hepatocytes accumulate huge amounts of lipids. Reduced oxygen supply, which also occurs during the development of this hepatic steatosis, as well as lipotoxicity due to lipid accumulation, both contribute to the development of cellular oxidative stress conditions. Consequently, at the end of this period of force-feeding, many markers of oxidative stress are radically modified but without any notable deregulation of their functionalities. This indicates that the hepatic cells are still actively coping with the metabolic challenge induced by the imposed feeding program. Nevertheless, the increased levels of activities of various caspases also indicate that these stressful conditions pushed an increased number of cells into the apoptotic cell death pathway.

In this experiment we demonstrate, for the first time, that oxidative stress sets in rapidly in the hepatocytes of ducks subjected to force-feeding. This could affect the normal functions of liver cells and it would be interesting to additionally test whether certain nutrients, such as natural antioxidants (vitamins C or E, beta-carotene, polyphenols, etc.) added in the diet before or during the force-feeding period, or synthetic products (Coenzyme Q10, glutathione, taurine, etc.) could attenuate this phenomenon (Supplementary Information).

## Supplementary Information

Supplementary Information.

## Data Availability

Details on the materials used are given in the M&M section. Raw data are available in the file data.xls for reviewers and on request to the corresponding author after publication.

## Abbreviations

ANOVA: ANalysis Of VAriance
B: Group of ducks at baseline, before the beginning of force-feeding
BFF: Group of BFF (Beginning of Force-Feeding) ducks
Cat: Catalase
CV: Coefficient of variation
DM: Dry matter
EFF: Group of EFF (End of Force-Feeding) ducks
ELISA: Enzyme-linked ImmunoSorbent assay
FFA: Free fatty acids
GSH: Reduced glutathione
GSSG: Oxidized glutathione
GSH/GSSG: Ratio GSH/GSSG
GPX1: Glutathione peroxidase 1
HIF1α: Hypoxia inducible factor 1 alpha
HIF2α: Hypoxia inducible factor 2 alpha
NAFLD: Non-alcoholic fatty liver disease
NASH: Non-alcoholic SteatoHepatitis
NQO1: Nicotinamide adenine dinucleotide (phosphate) hydrogen dehydrogenase quinone-1
OH-Pro: Hydroxy-PROline
Proc GLM: General linear model procedure
RMSE: Root mean square error
ROS: Reactive oxygen species
SD: Standard deviation
SOD: Super oxide dismutase
TBARS: Thio barbituric acid reactive substances
